# Improvement of conventional perception in stable patients with schizophrenia by add-on treatment with ipidacrine

**DOI:** 10.1192/j.eurpsy.2021.1408

**Published:** 2021-08-13

**Authors:** M. Morozova, S. Potanin, G. Rupchev, D. Burminskiy, T. Lepilkina, A. Beniashvili

**Affiliations:** Laboratory Of Psychopharmacology, FSBSI Mental Health Research Center, Moscow, Russian Federation

**Keywords:** ipidacrine, conventional perception, schizophrénia, add-on treatment

## Abstract

**Introduction:**

Impairment of conventional perception is one of the key dysfunction in patients with schizophrenia even in absence of psychotic symptoms.

**Objectives:**

Possibility of improvement of conventional perception by add-on treatment with ipidacrine in patients with schizophrenia in long-term remission.

**Methods:**

26 (13 females) patients, mean age 40.4 (SD 11.7) with episodic schizophrenia in remission more than one year, receiving stable antipsychotic therapy were included into the open label study. As add-on treatment ipidacrine was administered once per day in dosage 20 mg for two months. Positive and Negative Symptoms Scale (PANSS) was used to assess clinical symptoms and projective psychological method (Rorschach Test) was used to assess conventional perception.

**Results:**

The study showed that ipidacrine in a low dosage, added to standard antipsychotic treatment, was effective in relation to negative symptoms (PANSS negative subscale score before 22,4 (SD4,7) and after beginning of the study 19,7 (4,5), p=0, 001). Of all the indicators of the Rorschach test, there was significant improvement in the index X+%, which is responsible for the degree of conventionality in reality recognition. The decrease in conventionality was associated with both high individualism and perceptual disorders. The value of of X+ % did not reach the standard one (70%) to the end of the study, but the improvement showed the switching from severe (52,4 (SD 12,2) to moderate (60,6 (SD10,4) impairment level (p=0,039).

**Conclusions:**

Ipidacrine in a low dosage as add-on treatment has positive effect on conventional perception of stable patients with schizophrenia even in short-term trial.

